# Detergent-insoluble PFN1 inoculation expedites disease onset and progression in PFN1 transgenic rats

**DOI:** 10.3389/fnins.2023.1279259

**Published:** 2023-09-25

**Authors:** Shiquan Cui, Tingting Zhang, Xinrui Xiong, Jihe Zhao, Qilin Cao, Hongxia Zhou, Xu-Gang Xia

**Affiliations:** ^1^Department of Environmental Health Sciences, Robert Stempel College of Public Health & Social Work, Florida International University, Miami, FL, United States; ^2^Burnett School of Biomedical Sciences, University of Central Florida College of Medicine, Orlando, FL, United States; ^3^The Center for Translational Sciences, Florida International University, Miami, FL, United States

**Keywords:** PFN1, ALS, rat, DNAJB6, prion-like property, protein seeding, aggregation, neurodegeneration

## Abstract

Accumulating evidence suggests a gain of elusive toxicity in pathogenically mutated PFN1. The prominence of PFN1 aggregates as a pivotal pathological hallmark in PFN1 transgenic rats underscores the crucial involvement of protein aggregation in the initiation and progression of neurodegeneration. Detergent-insoluble materials were extracted from the spinal cords of paralyzed rats afflicted with ALS and were intramuscularly administered to asymptomatic recipient rats expressing mutant PFN1, resulting in an accelerated development of PFN1 inclusions and ALS-like phenotypes. This effect diminished when the extracts derived from wildtype PFN1 transgenic rats were employed, as detergent-insoluble PFN1 was detected exclusively in mutant PFN1 transgenic rats. Consequently, the factor influencing the progression of ALS pathology in recipient rats is likely associated with the presence of detergent-insoluble PFN1 within the extracted materials. Noteworthy is the absence of disease course modification upon administering detergent-insoluble extracts to rats that already displayed PFN1 inclusions, suggesting a seeding rather than augmenting role of such extracts in initiating neuropathological changes. Remarkably, pathogenic PFN1 exhibited an enhanced affinity for the molecular chaperone DNAJB6, leading to the sequestration of DNAJB6 within protein inclusions, thereby depleting its availability for cellular functions. These findings shed light on a novel mechanism that underscores the prion-like characteristics of pathogenic PFN1 in driving neurodegeneration in the context of PFN1-related ALS.

## Introduction

Mutation in profilin 1 (*PFN1*) is a pivotal factor in the etiology of amyotrophic lateral sclerosis (ALS) (Wu et al., 2012; Smith et al., 2015), although the precise mechanisms of motor neuron degeneration in this context remain elusive. Accumulated evidence from diverse animal models collectively posits a gain of pathogenic functionality inherent to mutated PFN1. Intriguingly, the overexpression of mutant, but not wildtype (WT), human PFN1 in mice and rats recapitulates the cardinal manifestations of ALS, encompassing progressive motor neuron degeneration, skeletal muscle denervation atrophy, and resultant paralysis (Yang et al., 2016; Fil et al., 2017; Yuan et al., 2021; Yusuf et al., 2022). These studies, using transgenic rodent paradigms, underscore that the observed disease phenotypes emanate primarily from the specific pathogenic mutation in PFN1 rather than a mere augmentation of its expression. PFN1, as a key orchestrator of cytoskeletal dynamics, cellular motility, endocytic processes, and synaptic integrity, assumes a critical role in fundamental neuronal functions (Witke, 2004; Taylor et al., 2016). Compellingly, the ablation of PFN1 in murine knockout (KO) models engenders developmental anomalies (Witke et al., 2001); however, the deficiency of PFN1 in mature mice does not culminate in appreciable neuropathological sequelae (Görlich et al., 2012). This intriguing disparity suggests that while PFN1 is indispensable for proper developmental trajectories, the survival and functional maintenance of mature neurons may be sustained through alternative compensatory mechanisms, dissociating mature neuronal viability from obligatory PFN1 presence. Studies using reverse genetic approaches suggest a gain of elusive toxicities in pathogenically mutated PFN1.

Protein aggregation is a prominent pathology detected prior to neurodegeneration in PFN1 transgenic rats (Yuan et al., 2021), and is a shared hallmark of neurodegenerative diseases like ALS (Neumann et al., 2006; Kwong et al., 2007, 2008), Alzheimer’s disease (Bruggink et al., 2012), and Parkinson’s disease (Goedert et al., 2012). Aggregated proteins can exert toxicity (Bucciantini et al., 2002; Walsh et al., 2002), disrupting cellular protein clearance systems and compromising vital functions. While the precise mechanisms of protein aggregation-induced neurodegeneration are under scrutiny (Spillantini et al., 1997; Luk et al., 2012a,b; Rodriguez et al., 2015), alpha-synuclein’s pivotal role in Parkinson’s pathology is evident from studies involving intracerebral injections of brain extracts from alpha-synuclein transgenic mice, which induced aggregation and neuropathology in recipients (Luk et al., 2012a,b). In ALS, the contribution of protein aggregation to disease initiation and progression remains less understood, despite focused investigations into protein inclusion formation and its impact on cellular function (Neumann et al., 2006; Polymenidou and Cleveland, 2011; Liu et al., 2012).

To address the compelling inquiry into the pathomechanisms of ALS, we examined the impact of inoculating PFN1 inclusions on the initiation and progression of neurodegeneration in PFN1 transgenic rats. The inoculation of a specific detergent-insoluble extract from diseased rat spinal cords *via* intramuscular administration expedited the onset and advancement of the disease. This acceleration was attributed to the facilitation of PFN1 inclusion formation in recipient rats expressing mutant human PFN1 at a young age. Pathogenic PFN1 displayed a heightened propensity for aggregation and an increased affinity for the molecular chaperone DNAJB6, leading to the sequestration of soluble DNAJB6 within protein inclusions. These findings underscore the important role of PFN1 aggregate formation in initiating ALS-related neurodegeneration.

## Materials and methods

### Animal study approval

Animal use was in accord with NIH guidelines and the animal use protocol was approved by the Institutional Animal Care and Use Committees at the University of Central Florida and at Florida International University.

### Animal experiments and immunohistochemistry

PFN1-C71G-V5 transgenic mice were purchased from Jackson laboratory (Yang et al., 2016) (stock No: 028608). Mice were euthanized and tissues were collected for immunoprecipitation at arrival.

PFN1 transgenic rats were created in the lab and maintained on a Sprague Dawley genomic background (Zhou et al., 2009). Rats were subjected to Open-Field Activity assay (Med Associates) and their motor function was assessed and monitored as previously described (Huang et al., 2012; Tong et al., 2013; Yuan et al., 2021). Disease onset manifested as an irreversible decline in the distance traveled, as documented by Open-Field activity assay, while paralysis was characterized by observable leg-dragging or an incapacity for leg retraction. The disease end-stage was denoted by the incapacitation to retract two or more legs, coupled with an inability to self-right when the rat was positioned laterally. Tissues were harvested subsequent to the euthanizing of transgenic rats.

Immunohistochemistry was performed on the cross sections of rat spinal cord and were examined of protein immunoreactivity with a Nikon microscope as previously described (Yuan et al., 2021).

### Extraction and inoculation of detergent-insoluble protein complex

The detergent-soluble and -insoluble fractions of tissue and cell homogenates were extracted using ultracentrifugation as previously described (Wu et al., 2015; Yuan et al., 2021). In brief, rat whole spinal cords or cultured cells were sonicated in a RIPA buffer (1% Triton X-100, 0.1% SDS) to release cellular contents at a ratio of 1 g tissues to 10 mL lysis buffer. Lysates were then centrifuged (100,000 x g, 10 min) to separate detergent-soluble and insoluble pellet fractions. Pellets were washed twice in cold PBS of the same volume as lysis buffer initially used and centrifuged again to remove detergent-soluble materials. For immunoblotting assay, final pellets were sonicated in lysis buffer containing 1% SDS and were boiled to dissolve precipitated proteins. For intramuscular injection, the final pellets derived from the 100 mg tissues of either WT or mutant *PFN1* transgenic rats were suspended and sonicated in 1 mL of sterile PBS to produce the suspension solution. For each rat, both hind legs were injected with 100 μL suspension solution into quadriceps femoris and gastrocnemius muscles.

### Cell culture and immunoblotting

HEK293 cells were grown in DMEM medium supplemented with 10% FBS and transfected of plasmids using Lipofectamine 2000 as described (Wu et al., 2015). Transfection occurred without FBS for 4 h, then FBS was added. Plasmids expressing a desired gene were sequence-verified before use. Control cells were sham-transfected with a plasmid expressing no mammalian genes. Cells were harvested for biochemistry assays 48 h after transfection.

For immunoblotting, animal tissues and HEK293 cells were homogenized in RIPA buffer. Proteins in lysates were separated using SDS-PAGE and transferred onto nitrocellulose membranes, and immunoreactivity for specific protein was detected using specific antibodies as previously described (Yuan et al., 2021).

### Immunoprecipitation and protein identification

For immunoprecipitation, cells and tissues were initially lysed in lysis buffer (Promega) and were fully broken by sonication as described (Xia et al., 2013). The lysates were cleared by centrifugation 10 min at 4°C and 500 μg of total protein per sample was incubated with V5 (Abcam), FLAG (Sigma), or MYC (ThermoFisher) binding resin to precipitate tagged proteins and affiliated protein partners. Bound proteins were eluted with SDS sample buffer and boiled for 10 min to dissociate protein complexes. Eluted proteins were detected by immunoblotting with specified antibodies. For protein identification, V5 resin beads were washed with PBS solution and proteins were eluted and precipitated before labeling with Cy2 or Cy3 dye. Fluorescent dye-labeled proteins were resolved on 2-dimenional gels and protein spots with increased intensity in PFN1 transgenic mouse tissues were picked up by a robot and were analyzed by mass spectrometry. Mass spectrometry and 2-D gel analyses were performed by Applied Biomics Inc. (CA, United States) as described (Bi et al., 2013).

### Antibody information

The immunoreactivity of a desired protein was detected with a specific primary antibody that was purchased from a verified resource: mouse monoclonal anti-GAPDH (Abcam, cat# ab8245), mouse monoclonal anti-FLAG (Proteintech, cat# 66008-4-Ig), mouse monoclonal anti-Myc (ThermoFisher, cat # MA1-980), rabbit polyclonal antibody against p62 (Novus, cat# NBP1-48320), rabbit anti-DNAJB6 (Proteintech, cat# 11707-1-AP), rabbit anti-ACTG1 (Proteintech, cat# 11227-1-AP), and rabbit anti-PFN1 (ThermoFisher, cat# PA5-17444). Primary antibodies were diluted at 1:1,000 for immunoblotting and were used for immunostaining at the lowest dilutions recommended by the manufacturers.

### Statistical analysis

Unpaired *t*-tests were used to analyze any differences in immunoblotting density between groups using Graphpad Instat software. A *p* value <0.05 was considered statistically significant. The data were not assessed for normality and no test for outliers was conducted.

## Results

### Intramuscular injection of insoluble spinal cord extract accelerated disease onset and shortened survival in PFN1 transgenic rats

**Figure 1:**
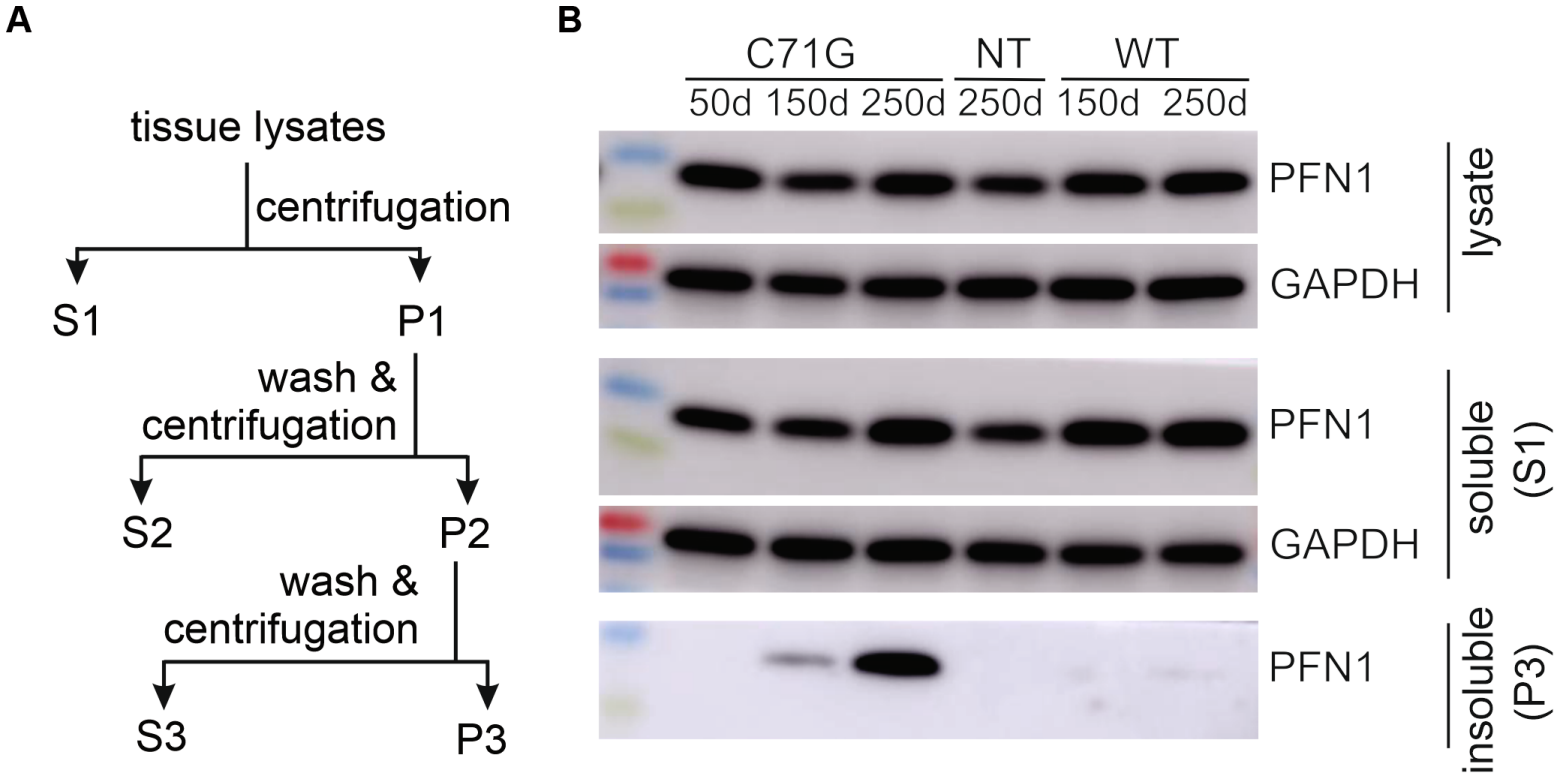
Detergent-insoluble fractions were extracted from the spinal cords of PFN1 transgenic rats. **(A)** A diagram shows the procedure of extracting detergent-insoluble fraction from TG rats expressing human PFN1 with or without the pathogenic mutation C71G. The whole spinal cords were harvested from euthanized rats and homogenized in tissue lysis buffer containing 1% Triton X-100 and 0.1% SDS. Tissue lysates were centrifugated at 100,000 g for 10 min. The first supernatant (S1) was considered as the detergent-soluble fraction, and the first pellet (P1) was subjected to washing in PBS and further ultracentrifugation to generate pellet 2 (P2). P2 was washed in PBS and centrifugated again to generate the third pellet (P3). P3 was considered the detergent-insoluble fractions. P3 was dissolved in lysis buffer containing 1% SDS for immunoblotting or dissolved in PBS by sonication for intramuscular injection. **(B)** Immunoblotting confirmed that the detergent-insoluble fractions contained the mutant, but not the WT, human PFN1 extracted from the spinal cord of PFN1 transgenic rats.

**Figure 2:**
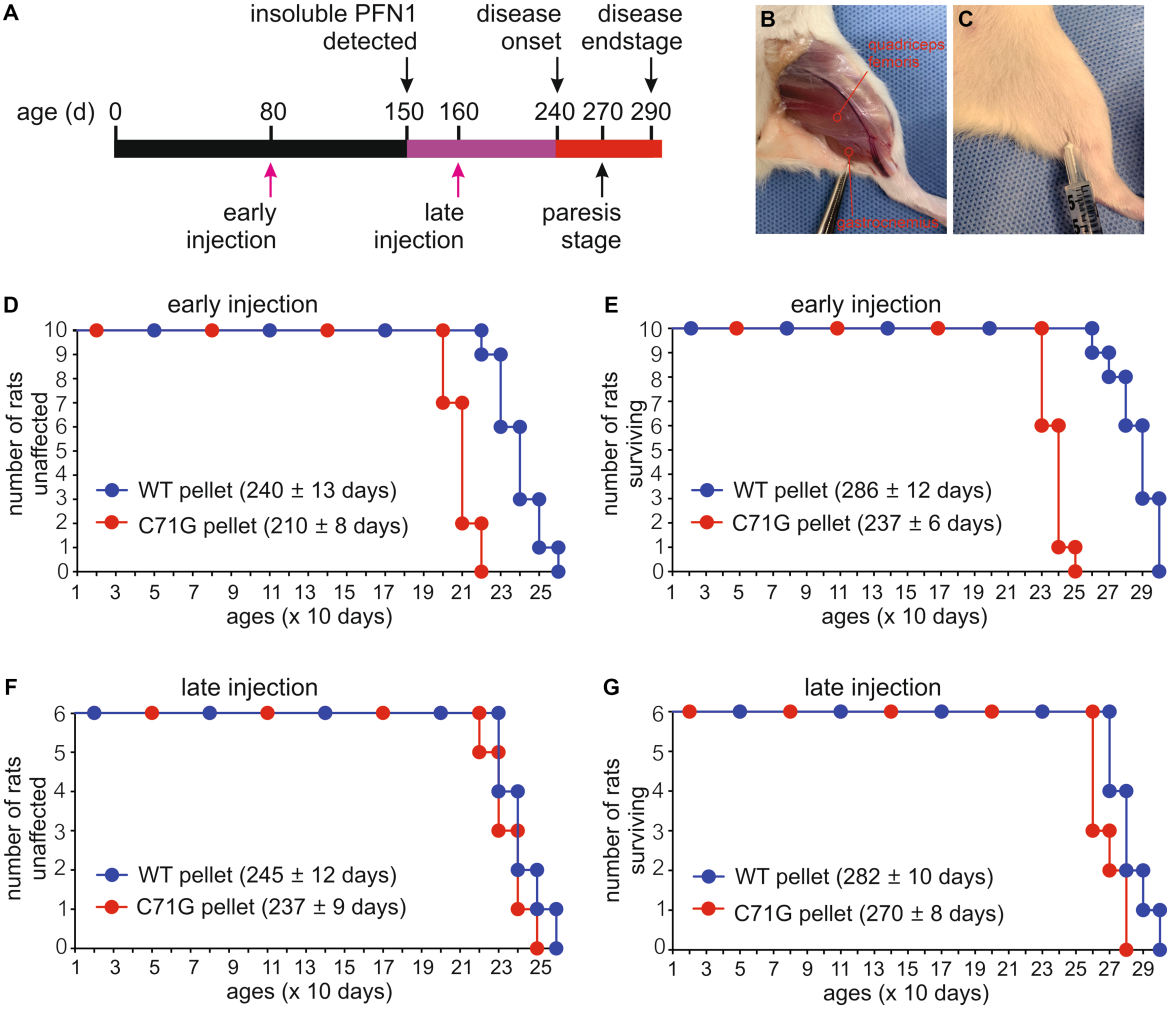
Intramuscular injection of protein inclusions accelerated disease onset and progression in *PFN1* transgenic rats. **(A)** A diagram showing the timeline of disease progression in mutant PFN1 transgenic (TG) rats and the time points for intramuscular injections of detergent-insoluble materials extracted from the spinal cords of WT or mutant PFN1 TG rats. **(B)** An image showing two selected sites for injecting insoluble materials into quadriceps femoris and gastrocnemius muscles. **(C)** Each of the two selected sites was injected with 100 μL of detergent-insoluble materials extracted from whole spinal cords of TG rats. **(D–G)** Probabilities of disease onset **(D,F)** and rat mortality **(E,G)** in TG rats injected with detergent-insoluble materials at 80 days old (early injection) or 160 days old (late injection). Detergent-insoluble materials were extracted from the spinal cords of age-matched WTs (WT pellet) and mutant (C71G pellet) PFN1 TG rats at paresis stage. Onset was determined by a non-recoverable decrease in mobility during the open-field test, and full paralysis determined mortality. Data represent mean ± s.d. (*n* = 10 or 6).

PFN1 aggregate is a predominant pathology detected long before the occurrence of paralysis in PFN1 TG rats (Yuan et al., 2021), suggesting that PFN1 aggregation is an important contributor rather than a bystander in motor neuron degeneration. To examine how PFN1 aggregation is involved in the pathogenesis of ALS, we extracted detergent-insoluble materials from the spinal cords of PFN1-C71G transgenic rats with paresis and suspended the precipitates in sterilized PBS by sonication (Figure 1). The intent was to assess how a seeding effect of insoluble PFN1 complex might impact the initiation and progression of neurodegeneration in ALS rats. To minimize the influence of surgery on phenotypic expression, we chose intramuscular administration over intraspinal administration of the insoluble PFN1 complex, even though this more remote administration may have reduced the seeding effect. Intramuscular injection has been shown to be effective for delivery of synthetic alpha-synuclein fibrils (Sacino et al., 2014), but this approach has not been tested for the delivery of insoluble tissue extracts. We tested the seeding effect of the insoluble spinal cord extract at two time points: at 80 days when detergent-insoluble PFN1 was undetectable, and at 160 days when it was (Figures 2A–C). We also extracted detergent-insoluble materials from the spinal cords of age-matched WT PFN1 transgenic rats and used the normal extract as a control for insoluble mutant PFN1 complex. Intriguingly, the earlier injection of mutant extract accelerated the onset and progression of the ALS phenotype in PFN1 transgenic rats (Figures 2D–E). When the insoluble PFN1 complex was administered to transgenic rats, disease onset was accelerated by 30 days on average (210 ± 8 vs. 240 ± 13, *p* < 0.05) and rat survival was shortened by 49 days (237 ± 6 vs. 286 ± 12, *p* < 0.05). By contrast, later injections did not show any significant modification of the disease course though a trend for facilitated disease course was also observed (Figures 2F–G). We paired mutant PFN1 transgenic rats from the same litters for intramuscular injections of either WT or mutant PFN1 extracts, minimizing the impact of rat genomic background on disease course. Intramuscular injections of insoluble PFN1 complex facilitated both the onset and progression of ALS disease in mutant PFN1 transgenic rats.

### Intramuscular injection of insoluble spinal cord extract produced a seeding effect on protein aggregation in PFN1 transgenic rats

**Figure 3:**
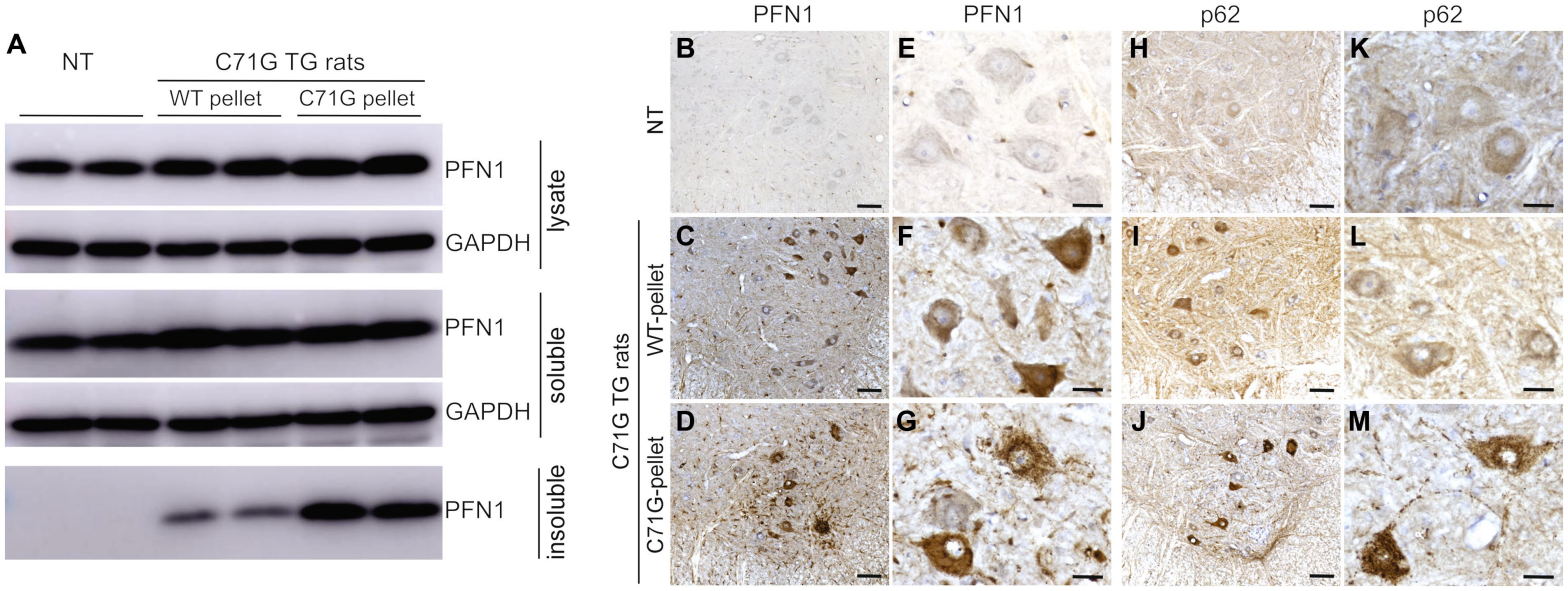
Intramuscular injection of protein inclusions facilitated protein aggregation in *PFN1* transgenic rats. **(A)** Biochemical analyses revealed that intramuscular injections of detergent-insoluble materials facilitated PFN1 aggregation in transgenic (TG) rats. Detergent-insoluble material was extracted from spinal cords of mutant or WT PFN1 TG rats at paresis, or matched ages, and was suspended in PBS. Mutant PFN1 TG rats (C71G) were euthanized at 150 days old when detergent-insoluble PFN1 was just detectable. **(B–M)** Immunohistochemistry revealed PFN1 **(B–G)** and p62 **(H–M)** inclusions in mutant PFN1 TG rats injected with C71G pellet solution. C71G TG rats and non-transgenic littermates (NT) were analyzed at 200 days old. Scale bars: 50 μm **(B–D,H–J)** and 30 μm **(E–G,K–M)**.

Reduced protein solubility is a common feature of all PFN1 mutations examined in our prior study (Yuan et al., 2021). Both WT and mutant PFN1 transgenic rats overexpress human PFN1 protein at comparable levels, but only mutant rats express a mutant form of PFN1 protein (Yuan et al., 2021). Spinal cord extracts from mutant PFN1 transgenic rats contained detergent-insoluble PFN1 that was not detected in the extracts from WT PFN1 transgenic rats (Figure 1). We hypothesized that intramuscular injections of detergent-insoluble materials from diseased rats would likely have a seeding effect on PFN1 aggregation. Biochemical analyses revealed that the formation of detergent-insoluble PFN1 complex was facilitated by the intramuscular injections of spinal cord extract from diseased rats (Figure 3). By 150 days of age, insoluble PFN1 was just detectable in mutant PFN1 transgenic rats that were injected with spinal cord extract from WT PFN1 transgenic rats (Figure 3A). In contrast, insoluble PFN1 was abundant in mutant transgenic rats that were injected with spinal cord extract from diseased rats (Figure 3A). Accelerated protein aggregation was confirmed by immunostaining of lumbar spinal cord cross sections (Figures 3B–M). Intramuscular injections of insoluble PFN1 complex therefore had a seeding effect on the aggregation of mutant PFN1 in transgenic rats.

### Pathogenic mutation increased the binding affinity of PFN1 for the molecular chaperone DNAJB6

**Figure 4:**
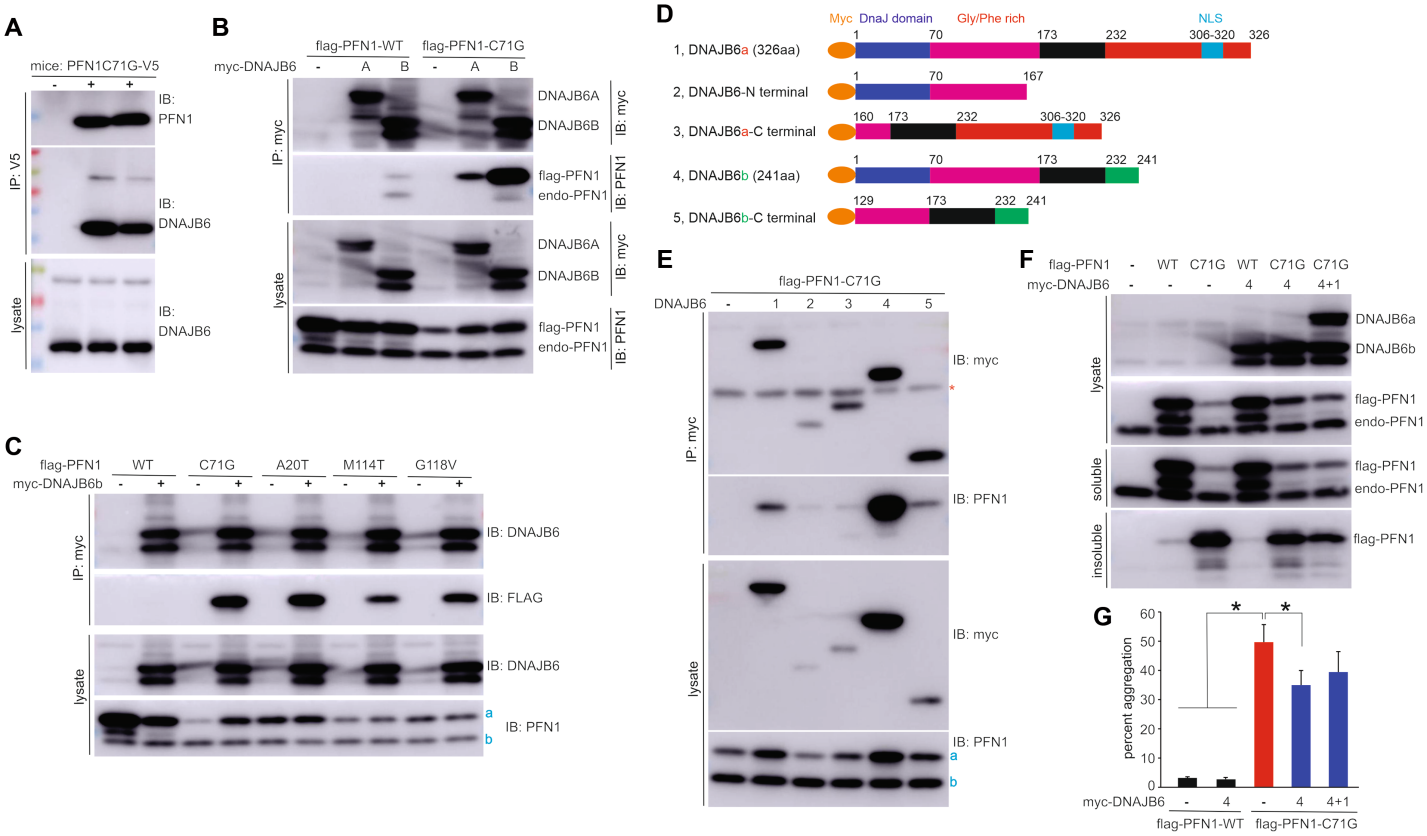
PFN1 with pathogenic mutation gains increased affinity for DNAJB6 to enhance its solubility. **(A)** Immunoprecipitation (IP) revealed that pathogenic PFN1 interacted with DNAJB6 in the lysates extracted from the forebrain of TG mice expressing V5-tagged human PFN1 with the pathogenic mutation C71G substitution. **(B,C)** IP detected that PFN1 mutants gained an increased affinity for DNAJB6 as compared to PFN1 wildtype (WT). HEK293 cells were transfected with plasmids expressing the genes indicated and DNAJB6 complexes were pulled down with MYC beads on which existence of PFN1 was revealed by immunoblotting (IB). **(D)** Schematics showing the fragments of DNAJB6 isoforms A (DNAJB6a) and B (DNAJB6b) used for IP to detect the domains interacting with PFN1. **(E)** IP detected that PFN1 mutant interacted mainly with full-length DNAJB6b and minimally with full-length DNAJB6a. Note that both DNAJB6a and DNAJB6b enhanced mutant PFN1 stability in HEK293 cells. **(F,G)** Overexpression of DNAJB6b reduced aggregation of mutant PFN1. HEK293 cells were transfected with plasmids expressing FLAG-tagged PFN1 and MYC-tagged DNAJB6. Detergent-soluble and -insoluble fractions derived from 1 ug of total proteins in cell lysates were examined of PFN1 immunoreactivity and percent PFN1 aggregation was thus calculated. Data were means + SD (*n* = 5). * *p* < 0.05. In panels **(C,E)**: a, flag-tagged PFN1; b, endogenous PFN1.

To unravel the molecular pathways leading to neurodegeneration in PFN1-caused disease, we attempted to identify the protein partners interacting preferably with pathogenic PFN1. We purified mutant PFN1 protein complex from the brain of transgenic mice carrying human PFN1-C71G (Yang et al., 2016). As the PFN1-C71G protein was tagged with V5 at the N-terminal, mutant PFN1 protein complex was purified with V5 resin and the immunoprecipitates were resolved on 2-dimensional gel (Supplementary Figure S1). Protein spots found in V5-PFN1-C71G transgenic mice and not in non-transgenic control mice were picked up by a robot and were examined of protein components by mass spectrometry. Actin gamma 1 (ACTG1) and DnaJ homolog subfamily B member 6 (DNAJB6) were identified as protein partners of mutant PFN1 by mass spectrometry and were confirmed by immunoblotting (Figures 4; Supplementary Figures S1, S2). ACTG1 is an established interactor of PFN1 and served as a positive control for PFN1 immunoprecipitation (Korenbaum et al., 1998; Hajkova et al., 2000).

Two isoforms of DNAJB6 (DNAJB6A & DNAJB6B) are expressed in mammalian cells with differentiated expression patterns in the nucleus and the cytoplasm (Sarparanta et al., 2012), respectively. We assessed the effect of pathogenic mutation on the binding affinity of PFN1 for DNAJB6. Mutant PFN1-C71G was pulled down preferably by the cytoplasmic isoform DNAJB6B and minimally by the nuclear isoform DNAJB6A (Figure 4B). Interaction between DNAJB6 and WT PFN1 was barely detected (Figure 4B). We examined three other ALS-causing PFN1 mutations (i.e., A20T, M114T & G118V) in cultured cells and observed increased binding to DNAJB6 for all the PFN1 mutations assessed (Figure 4C). Pathogenic mutation afforded PFN1 an increased affinity for the molecular chaperone DNAJB6. We split DNAJB6 into the N-terminal and the C-terminal domains to determine the binding sites on DNAJB6 for PFN1 and found that PFN1 mainly interacted with the full-length DNAJB6B (Figures 4D,E). A minimal interaction between mutant PFN1 and DNAJB6 fragments was detected (Figure 4E). Overexpression of DNAJB6B in cultured cells reduced the aggregation of mutant PFN1 (Figures 4F,G), suggesting that mutant PFN1 requires increased assistance in folding.

### Pathogenic PFN1 depleted soluble DNAJB6 by precipitating the chaperone in protein inclusions

**Figure 5:**
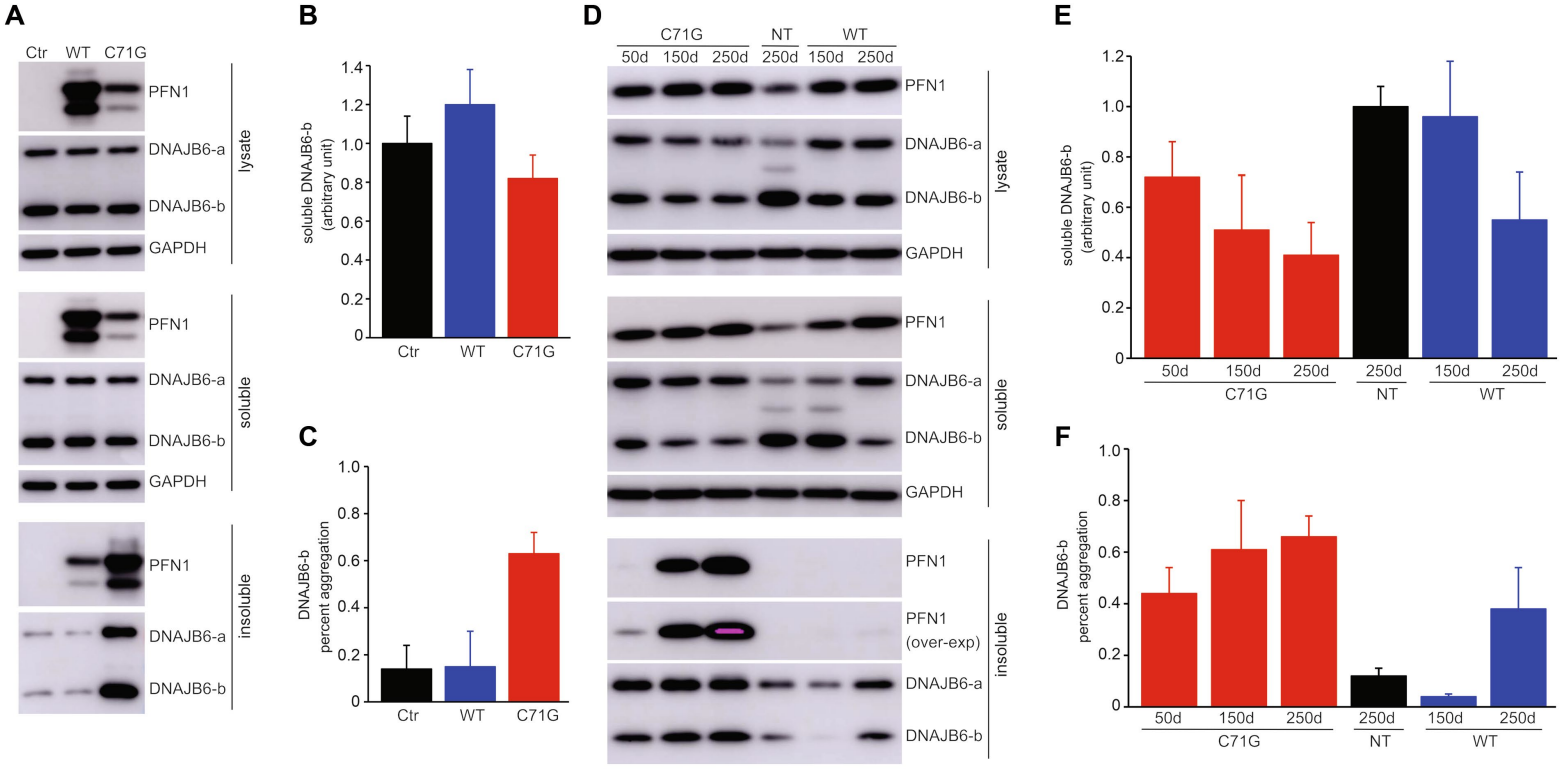
Pathogenic PFN1 traps DNAJB6 in protein inclusions to deplete DNAJB6 from soluble fractions. **(A–C)** Overexpression of mutant (C71G), but not wildtype (WT), PFN1 reduced DNAJB6 solubility by precipitating it in protein aggregates. HEK293 cells were transfected with plasmids expressing WT or C71G PFN1 and the cell lysates were fractionated into detergent-soluble and -insoluble fractions. DNAJB6b immunoreactivity in the soluble fraction was normalized to GAPDH and was calculated as the ratio of transfected cells to non-transfected cells **(B)**. Percent aggregation of DNAJB6b was calculated as the ratio of insoluble DNAJB6b to both soluble and insoluble DNAJB6b. Data were means + SD (*n* = 5). **(D–F)** Expression of pathogenic PFN1 in transgenic (TG) rats progressively depleted soluble DNAJB6b in the spinal cords. Soluble DNAJB6b and percent DNAJB6b aggregation in the lysates of rat spinal cords were calculated similarly to those in transfected HEK293 cells **(A–C)**. Data were means + SD (*n* = 3).

As pathogenic mutation of PFN1 increased its affinity for DNAJB6 and afforded PFN1 a propensity to aggregation (Figures 1, 4), we attempted to determine how pathogenic PFN1 affects the solubility of DNAJB6. Cells transfected with mutant, but not wildtype, human PFN1 displayed a large fraction of DNAJB6 coexisted with mutant PFN1 in detergent insoluble fraction (Figures 5A–C). Initial finding in cultured cells motivated us to examine the effect of mutant PFN1 on DNAJB6 in transgenic rats. As disease was progressing in PFN1-C71G transgenic rats, both PFN1 and DNAJB6 accumulated in detergent insoluble fractions, leading to a significant decrease in soluble DNAJB6 (Figures 5D–F). Pathogenic PFN1 gained an increased affinity for DNAJB6 and precipitated the molecular chaperone in aggregates, leading to depletion of soluble DNAJB6 in cells affected.

## Discussion

The initial identification of detergent-insoluble PFN1 as a primary pathological hallmark within PFN1 transgenic rats prompted a hypothesis that the process of PFN1 inclusion formation could potentially play a central role in the early stages of neurodegeneration in PFN1 transgenic rats (Yuan et al., 2021). Subsequent investigation, involving the intramuscular administration of detergent-insoluble extracts obtained from paralyzed rats, uncovered an accelerated development of PFN1 inclusions and ALS-like phenotypes in recipient rats that had previously displayed no symptom, yet carried mutant PFN1 transgene. This alteration in the disease trajectory of recipient rats can be ascribed to the molecular constituents embedded within the insoluble materials extracted from diseased rats that expressed the mutant PFN1 protein. Although both WT and mutant PFN1 transgenic rats demonstrated comparable levels of human PFN1 expression, the presence of detergent-insoluble PFN1 was observed exclusively in the spinal cord extract derived from paralyzed mutant PFN1 transgenic rats. As a result, it is reasonable to infer that the factor influencing the progression of ALS disease in recipient rats is likely associated with detergent-insoluble PFN1 present within the extracted materials.

The effectiveness of intramuscular administration to deliver protein oligomers is exemplified by the intramuscular injection of synthetic alpha-synuclein fibrils and brain homogenates (Sacino et al., 2014). Detergent-insoluble protein complexes are internalized by motor neuron terminals and subsequently retrogradely transported to the cell soma, thereby triggering protein aggregation process. In comparison to synthetic fibril administration, tissue extracts containing a lower load of pathological protein confer a moderate yet appreciable impact (Luk et al., 2012a,b). Noteworthy is the absence of disease course modification upon administering detergent-insoluble extracts to rats that already displayed PFN1 inclusions, suggesting a seeding rather than augmenting role of such extracts in the initiation of neuropathological changes. These findings elucidate a new underlying mechanism, underscoring the prion-like properties of pathogenically mutated PFN1 in propelling the onset and advancement of neurodegeneration in this disease.

**Figure 6:**
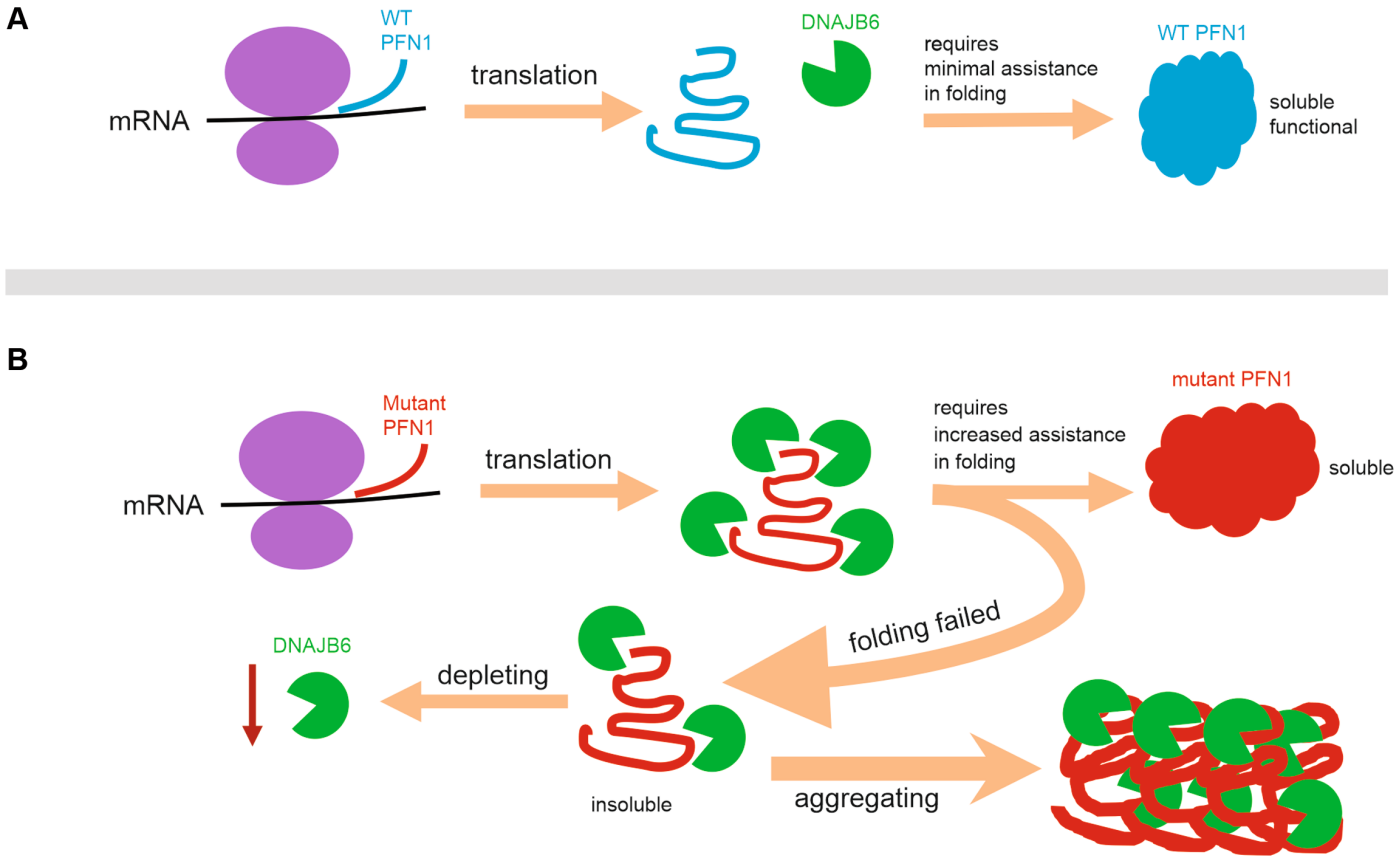
Schematic diagram illustrating a mechanism by which pathogenic PFN1 depletes DNAJB6b to compromise cellular functions. **(A)** Wildtype PFN1 requires minimal assistance in folding and possesses a good solubility. **(B)** Pathogenic PFN1 gains a pro-aggregation property and requires increased assistance in folding, leading to precipitation of molecular chaperones such as DNAJB6b in protein aggregates and thus to depletion of functional DNAJB6b to compromise cellular functions.

The general propensity for PFN1 mutations to aggregate agrees with ALS severity in patients (Wu et al., 2012; Smith et al., 2015). Mutant PFN1 may undergo a dynamic process of assembling and disassembling into detergent-insoluble inclusions. This phenomenon of protein aggregation, in and of itself, holds the potential for eliciting deleterious effects (Bucciantini et al., 2002; Walsh et al., 2002). The aggregation of abnormal proteins may impede the efficacy of intracellular degradation machinery or sequester pivotal proteins, consequently compromising fundamental cellular operations (Wong and Holzbaur, 2014; Deng et al., 2019). Pathogenically mutated PFN1 gained an increased affinity for the molecular chaperone DNAJB6, the heightened propensity of mutant PFN1 that engendered the sequestration of DNAJB6 within protein inclusions and thereby culminated in the depletion of soluble DNAJB6 within the cellular milieu. Insoluble proteins are deemed to be inaccessible for cellular function (Pallares and Ventura, 2016; Mogk et al., 2018). The diminution of soluble DNAJB6 is likely to curtail the availability of this critical molecular chaperone to fulfill its cellular functions, thereby potentially causing injurious consequences upon the afflicted cells. The indispensable role of DNAJB6 in cellular function is underscored by the finding that its deficiency, as evidenced in murine KO models, engenders aberrations in neural stem cell self-renewal process (Watson et al., 2009). Moreover, the upregulation of DNAJB6 has been shown to confer neuroprotection against the toxic ramifications of aggregated polyglutamine or alpha-synuclein (Kakkar et al., 2016; Aprile et al., 2017). Intriguingly, recessive mutation in DNAJB6 causes limb-girdle muscular dystrophy due to defected chaperone function in the protein (Sarparanta et al., 2012). As illustrated in the diagram (Figure 6), our results suggest that pathogenically mutated PFN1 gains an enhanced binding affinity with the molecular chaperone DNAJB6 and co-aggregates with DNAJB6 to form functionally inaccessible protein inclusions and thus to compromise DNAJB6 function. These findings unveil a new mechanistic insight into the pathomechanisms of PFN1-caused neurodegeneration and suggest that enhancing the molecular chaperone DNAJB6 may provide a protection against PFN1-caused neurodegeneration in the disease.

## Data availability statement

The original contributions presented in the study are included in the article/Supplementary material, further inquiries can be directed to the corresponding authors.

## Ethical statement

The animal studies were approved by Institutional Animal Care and Use Committees at the University of Central Florida and at Florida International University. The studies were conducted in accordance with the local legislation and institutional requirements. Written informed consent was obtained from the owners for the participation of their animals in this study.

## Author contributions

SC: Data curation, Formal analysis, Investigation, Methodology, Writing – review & editing. TZ: Data curation, Formal analysis, Investigation, Methodology, Writing – review & editing. XX: Data curation, Investigation, Methodology, Writing – review & editing. JZ: Conceptualization, Resources, Writing – review & editing. QC: Conceptualization, Writing – review & editing. HZ: Conceptualization, Funding acquisition, Supervision, Writing – original draft, Writing – review & editing. X-GX: Conceptualization, Funding acquisition, Supervision, Writing – original draft.

## Funding

The author(s) declare financial support was received for the research, authorship, and/or publication of this article. This work is supported by the National Institutes of Health (NIH)/National Institute of Neurological Disorders and Stroke (NS110455 and NS089701 to X-GX and HZ) and NIH/National Institute on Aging (AG064822 to X-GX. and HZ).

## Conflict of interest

The authors declare that the research was conducted in the absence of any commercial or financial relationships that could be construed as a potential conflict of interest.

## Publisher’s note

All claims expressed in this article are solely those of the authors and do not necessarily represent those of their affiliated organizations, or those of the publisher, the editors and the reviewers. Any product that may be evaluated in this article, or claim that may be made by its manufacturer, is not guaranteed or endorsed by the publisher.
