# Supplementary material for: Detergent-insoluble PFN1 inoculation expedites disease onset and progression in PFN1 transgenic rats
Source: Front Neurosci. 2023 Sep 25;17:1279259. doi: 10.3389/fnins.2023.1279259 (PMC10560758; doi:10.3389/fnins.2023.1279259)
Supplement: Supplementary file 1 [file Data_Sheet_1.docx]

**
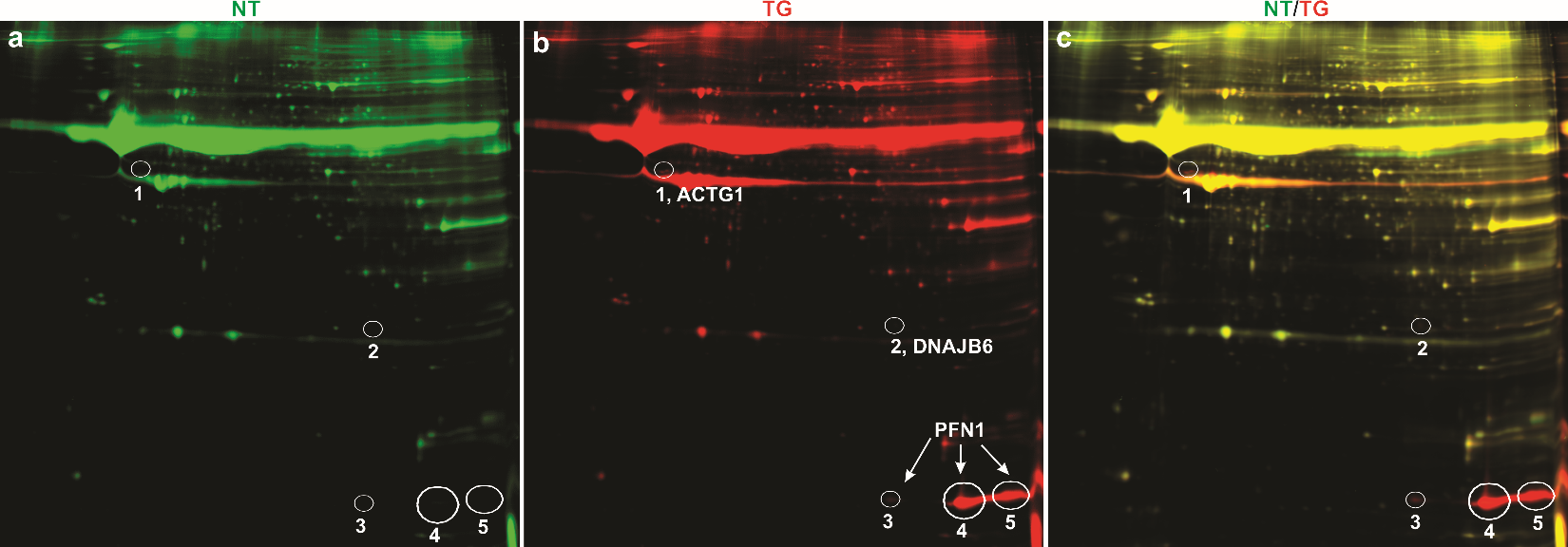
**

**Figure S1: Mass spectrometry combined with 2-dimensional gel assay revealed presence of DNAJB6 in PFN1 protein complexes. a-c)** PFN1 protein complexes were purified with V5 beads from the brain lysates of TG and NT mice. Proteins precipitated with V5 beads were labeled with green and red fluorescence dyes and resolved on 2-dimensional gels, and the protein spots of differentiated intensities between NT and TG were dissected from the gel and were examined of protein identifies by mass spectrometry. ACTG1 and DNAJB6 were revealed of their presence in purified PFN1 complexes.

**
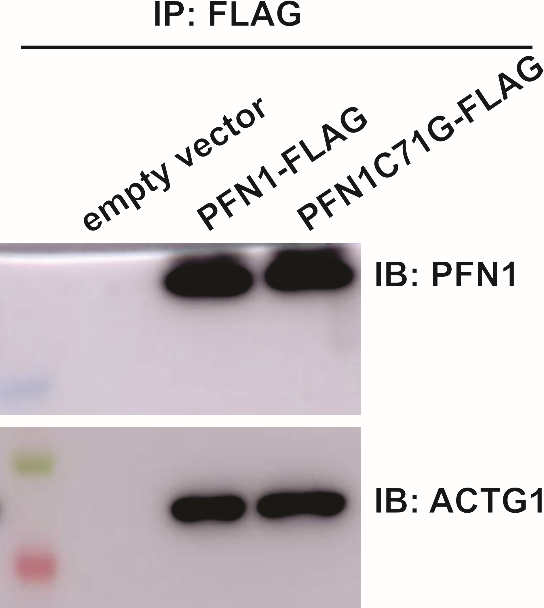
**

**Figure S2: ACTG1 was confirmed of its interaction with both wildtype and mutant PFN1.** HEK293 cells were transfected with plasmids expressing human PFN1 with or without the pathogenic mutation C71G substitution and PFN1 protein complexes were purified with FLAG beads. Presence of ACTG1 in PFN1 complex was confirmed by subsequent analysis of immunoprecipitates with an ACTG1 antibody.
